# Association of prothrombin complexe concentrate with venous thrombosis after cardiac surgery: a case-control study

**DOI:** 10.3389/fcvm.2023.1237227

**Published:** 2023-09-15

**Authors:** Qiaowei Zheng, Liting Zhang, Tingting Liu, Dong Guan, Weiyi Feng, Saisai Luo

**Affiliations:** Department of Pharmacy, The First Affiliated Hospital, Xi’an Jiaotong University, Xi’an, China

**Keywords:** prothrombin complex concentrate, cardiac surgery, venous thrombosis, case-control study, bleeding

## Abstract

**Background:**

Prothrombin complex concentrate (PCC) enhances coagulation and controls bleeding. We aimed to assess whether perioperative infusion of PCC is associated with venous thrombosis after cardiac surgery.

**Methods:**

We conducted a case-control study of patients undergoing cardiac surgery at our hospital in 2021. Multivariate logistic regression was used to assess the correlation between perioperative PCC infusion and postoperative venous thrombosis in cardiac surgery. Stratified analysis was also performed by age, hospitalization days, and whether warfarin, warfarin combined with heparin, warfarin combined with antiplatelet drugs were used postoperatively.

**Results:**

Data from 161 patients undergoing cardiac surgery were included in the analysis. Of these, 37 (23.0%) patients in the case group developed venous thrombosis, and 124 (77.0%) patients in the control group did not develop venous thrombosis. In the analysis without adjustment for confounders (model 1), perioperative PCC infusion significantly increased the risk of postoperative venous thrombosis (OR: 3.10, 95% CI: 1.26–7.59, *P *= 0.0135). In the model analysis adjusted for sex, age, and hospitalization days (model 2), perioperative PCC infusion was no longer significantly associated with the risk of postoperative venous thrombosis (OR: 1.76, 95% CI: 0.56–7.59, *P *= 0.3317). In the fully adjusted model (model 3), there was a marginally significant association between perioperative infusion of PCC and the risk of postoperative venous thrombosis (OR: 0.03, 95% CI: 0.00–1.23, *P *= 0.0637).

**Conclusions:**

Our findings show no significant association between perioperative PCC infusion in cardiac surgery and the development of postoperative venous thrombosis. Randomized controlled trials are needed to determine the causal relationship between perioperative PCC infusion and venous thrombosis in cardiac surgery.

## 1. Introduction

The occurrence of thrombotic events after cardiac surgery is a serious complication associated with adverse outcomes. However, the incidence of venous thrombosis in patients undergoing cardiac surgery is unclear. One study reported that the incidence of asymptomatic deep vein thrombosis (DVT) in patients undergoing cardiac surgery was 13% (1). Another study reported a 2.07% incidence of postoperative DVT in patients undergoing cardiac surgery from 2005 to 2010, with a risk of DVT twice that of patients undergoing vascular surgery and three times that of patients undergoing general surgery (2). A study showed that DVT occurred in 1.62% of approximately 30,000 patients undergoing cardiac surgery (3). Thromboembolism leads to increased mortality associated with cardiac surgery, the underlying mechanisms are multifactorial, such as disruption of coagulation homeostasis by the procedure itself, resulting in alterations such as venous blood flow stasis, endothelial damage, and abnormal coagulation mechanisms, but remain partially unclear. Epidemiological studies have identified many risk factors associated with the development of venous thrombosis (4), and studies showed that advanced age, coronary artery disease, dementia, renal disease, pulmonary disease, smoking, and fibrinogen are associated with venous thrombosis (5).

Patients with bleeding associated with cardiac surgery have varying degrees of coagulation dysfunction, and the mechanisms are multifactorial. This includes thrombocytopenia and platelet dysfunction, reduced fibrinogen concentration, impaired thrombin production, and depletion of coagulation factors (6). Patients undergoing cardiac surgery with extracorporeal circulation (CPB) have an average of 40%–50% decreased levels of coagulation factors and 50% decreased coagulation production (7). Overall, prolonged CPB and anticoagulant use in patients undergoing cardiac surgery predispose to reduced coagulation factor activity and enhanced fibrinolytic system, which increases the risk of bleeding during cardiac surgery (8, 9). The use of coagulation factors during cardiac surgery (10–13), particularly prothrombin complex concentrate (PCC), has increased over the last two decades. PCC contains coagulation factors II, VII, IX, and X as well as natural coagulation inhibitors protein C and S (14), which effectively stop bleeding while minimizing the risk of thrombosis. Most studies have focused on the effectiveness of PCC, but its safety is an important issue when it is used. Four larger observational studies have reported a potentially higher risk of acute kidney injury with PCC (10, 15–17). Historically, the main drawback of PCC has been the risk of thrombotic complications (18). In cardiac surgery, some studies have reported serious thromboembolic events with PCC (19–21). However, it is unclear whether perioperative use of PCC is associated with venous thrombosis after cardiac surgery.

Therefore, we aimed to evaluate the effect of perioperative use of PCC on postoperative venous thrombosis in patients undergoing cardiac surgery.

## 2. Materials and methods

### 2.1. Participants

This was a single-center, retrospective case-control study screening inpatients who underwent cardiac surgery at the First Affiliated Hospital of Xi'an Jiaotong University in 2021. The inclusion criteria of this study were: patients were >18 years old, coronary artery bypass grafting (CABG), valve or both with or without other procedures. The exclusion criteria were: Extracorporeal Membrane Oxygenation (ECMO) assisted patients, minimally invasive surgery or patients with a history of thrombosis. The case group consisted of patients who developed venous thrombosis after cardiac surgery until discharge, and the control group consisted of patients who did not develop venous thrombosis during the same period after cardiac surgery until discharge. Both our cases and controls were from inpatients undergoing cardiac surgery at our cardiovascular surgery department in 2021, reducing the possibility of selection bias.

Generally, after the neutralization of protamine during the operation, the attending physician administered empirically PCC based on the patient's intraoperative bleeding volume, extracorporeal circulation time, and thromboelastogram (TEG). In addition, whether PCC was used or not was consistent during this study. Perioperative monitoring and anesthesia management were determined by the attending anesthesiologist.

The project was approved by the Ethics Committee of the First Affiliated Hospital of Xi'an Jiaotong University.

### 2.2. Data collection

Clinical and laboratory data were collected from patients, including general patient characteristics, medical history, preoperative laboratory data, surgical characteristics and medication use, and postoperative characteristics. Patient characteristics included age, sex, days in hospital, and body mass index (BMI). Medical history data included smoking and drinking status, hypertension, diabetes, coronary artery disease, hyperlipidemia, atrial fibrillation, and tumors. Preoperative laboratory data included hemoglobin, red blood cell count, platelet count, activated partial thromboplastin time, D-dimer, total bilirubin, albumin, alanine aminotransferase, creatinine, etc. Surgical characteristics and medication use included total operative time, surgery type (isolated CABG, isolated valve, other), whether plasma, PCC, tranexamic acid, warfarin were used. Postoperative Characteristics included postoperative ICU time, re-exploration for bleeding, postoperative coagulopathy. The clinical and surgical data of all cases are sourced from our hospital's electronic medical record system.

Patients using PCC were divided into four groups according to the amount of PCC used: no use, low dose, medium dose, and high dose.

### 2.3. Study endpoints

The designated endpoint was the presence of venous thrombosis after cardiac surgery until the patient was discharged from the hospital. Venous thrombosis is detected by color Doppler ultrasonography, and recorded in the patient's electronic file.

### 2.4. Statistical analysis

Continuous variables are presented as Mean ± SD, and categorical variables are shown as *n* (%). Patients' demographic, and clinical characteristics data were analyzed using the Wilcoxon rank-sum test or the Pearson chi-square test. A multivariable logistic regression model was used to evaluate the relationship between PCC and venous thrombosis with the odds ratio (OR) and corresponding 95% confidence interval (CI), respectively. We constructed three models following the recommendations of the Strengthening the Reporting of Observational Studies in Epidemiology (STROBE) guidelines (22). In model 1, we did not adjust for variables. Model 2 is a crude model in which we adjusted only for sex, age, and hospitalization days. In model 3 we adjusted for variables that were statistically significant for univariate analysis and clinically significant variables.

We then explored the relationship between age and hospitalization days with venous thrombosis by smoothing curves, and we further applied a two-stage linear regression model to investigate the threshold effects of age and hospitalization days. The risk of perioperative PCC infusion and postoperative venous thrombosis was then analyzed stratified by age, hospitalization days, and whether warfarin, warfarin combined with heparin, warfarin combined with antiplatelet drugs were used postoperatively.

All analyses were conducted using EmpowerStats software and R version 4.2.0, with a *P* < 0.05 regarded as statistically significant.

## 3. Results

### 3.1. General information

During the year 2021, 161 patients were eventually enrolled in this study. There were 37 patients with venous thrombosis after cardiac surgery before discharge, randomly select 124 patients who did not experience venous thrombosis during the same period, and no Pulmonary embolism occurred in the case group and the control group. However, the mean age in the case group was 59.8 ± 11.6 years and in the control group was 52.6 ± 13.4 years, with a significant difference between the two groups. More patients in the case group had a history of hepatic and renal insufficiency and paraplegia, while patients in the control group had higher mean values of preoperative laboratory indices of platelet count, prothrombin time, activated partial thromboplastin time, total bilirubin, and albumin (Table 1).

**Table 1:** Characteristics of cases and controls.

	Cases (*n* = 37)	Controls (*n* = 124)	*P*
Baseline characteristics			
Sex (male)	22 (59.5%)	90 (72.6%)	0.128
Age (years)	59.8 ± 11.6	52.6 ± 13.4	0.004
Hospitalization days (d)	42.7 ± 18.3	18.8 ± 10.5	<0.001
BMI (kg/m^2^)	24.3 ± 5.0	23.7 ± 3.5	0.507
Medical history			
Hypertension	16 (43.2%)	52 (41.9%)	0.888
Diabetes mellitus	7 (18.9%)	23 (18.5%)	0.959
Hyperlipidemia	1 (2.7%)	0 (0.0%)	0.066
Atrial fibrillation	8 (21.6%)	21 (16.9%)	0.515
Tumor	3 (8.1%)	4 (3.2%)	0.201
Gastrointestinal bleeding	1 (2.7%)	0 (0.0%)	0.066
Smoking	11 (29.7%)	53 (42.7%)	0.156
Alcohol consumption	6 (16.2%)	27 (21.8%)	0.462
Pacemaker placement	0 (0.0%)	2 (1.6%)	0.437
History of fracture	1 (2.7%)	7 (5.6%)	0.47
Renal insufficiency	3 (8.1%)	1 (0.8%)	0.012
Liver insufficiency	3 (8.1%)	1 (0.8%)	0.012
Paraplegia	2 (5.4%)	1 (0.8%)	0.008
Central venous line placement	35 (94.6%)	92 (74.2%)	0.008
Preoperative laboratory variables			
HGB (g/L)	155.4 ± 156.3	132.5 ± 22.1	0.115
RBC (10^12^/L)	4.2 ± 1.0	4.4 ± 0.6	0.119
PLT (10^9^/L)	161.5 ± 64.9	192.8 ± 61.5	0.008
PT (s)	14.7 ± 4.9	13.3 ± 4.1	0.09
APTT (s)	34.5 ± 9.3	31.0 ± 6.9	0.015
INR	1.2 ± 0.5	1.1 ± 0.4	0.181
D-D (mg/L)	6.9 ± 16.4	2.0 ± 7.1	0.011
FIB (g/L)	3.3 ± 2.4	3.2 ± 1.2	0.629
TBIL (umol/L)	27.5 ± 29.4	15.8 ± 10.8	<0.001
IDBIL (umol/L)	19.2 ± 21.3	11.2 ± 7.6	<0.001
DBIL (umol/L)	8.3 ± 9.5	5.4 ± 7.7	0.055
ALB (g/L)	35.2 ± 4.3	38.5 ± 4.9	<0.001
CHOL (mmol/L)	3.7 ± 1.1	3.5 ± 0.9	0.494
ALT (U/L)	36.1 ± 48.5	139.7 ± 897.5	0.485
AST (U/L)	43.9 ± 58.1	125.2 ± 1,076.6	0.648
BUN (mmol/L)	8.4 ± 4.7	7.2 ± 4.1	0.154
CRE (umol/L)	89.8 ± 61.0	75.9 ± 68.7	0.269
Surgical characteristics and medication			
Total duration of surgery (min)	348.1 ± 133.9	258.4 ± 94.8	<0.001
Surgery type			0.536
Isolated CABG	5 (13.5%)	22 (17.7%)	
Isolated valve	13 (35.1%)	51 (41.1%)	
Other	19 (51.4%)	51 (41.1%)	
Total extracorporeal circulation intake (ml)	5,405.1 ± 1,661.2	4,152.7 ± 1,541.6	<0.001
Plasma (ml)	31 (88.6%)	90 (73.8%)	0.066
Red blood cells (U)	29 (82.9%)	70 (57.9%)	0.007
Usage of PCC			0.015
No use	7 (18.9%)	52 (41.9%)	
≥200–≤400 IU	18 (48.6%)	55 (44.4%)	
>400–≤1,000 IU	7 (18.9%)	12 (9.7%)	
>1,000–≤1,600 IU	5 (13.5%)	5 (4.0%)	
PCC	30 (81.1%)	72 (58.1%)	0.011
Heparin dosage (mg)	201.4 ± 96.9	189.3 ± 56.1	0.341
Protamine dosage (mg)	294.9 ± 139.3	271.6 ± 79.9	0.201
Tranexamic acid	21 (58.3%)	56 (45.5%)	0.176
Blood loss(ml)	266.7 ± 124.2	201.6 ± 139.4	0.013
Warfarin	17 (45.9%)	76 (61.3%)	0.097
Warfarin combined with heparin drugs	15 (40.5%)	44 (35.5%)	0.575
Warfarin combined with antiplatelet drugs	5 (13.5%)	8 (6.5%)	0.166
Postoperative characteristics			
Renal insufficiency	22 (61.1%)	26 (21.0%)	<0.001
Liver insufficiency	7 (19.4%)	5 (4.0%)	0.002
ICU days(d)	21.0 ± 19.3	4.1 ± 4.2	<0.001
Re-exploration for bleeding	2 (5.4%)	3 (2.4%)	0.358
Other thromboembolic events	6 (16.2%)	1 (0.8%)	<0.001
Postoperative coagulopathy	4 (10.8%)	2 (1.6%)	0.01
Total hospitalization expenses ($)	75,135.9 ± 39,048.5	25,466.4 ± 13,163.7	<0.001

Mean ± SD for continuous variables: The *p*-value was calculated by the weighted linear regression model.

% for categorical variables: *P*-value was calculated by weighted chi-square test.

BMI, body mass index; HGB, Hemoglobin; RBC, Red blood cell count; PLT, Platelet count; PT, Prothrombin time; APTT, activated partial thromboplastin time; INR, international normalized ratio; D-D, D-dimer; FIB, fibrinogen; TBIL, total bilirubin; IDBIL, indirect bilirubin; DBIL, direct bilirubin; ALB, albumin; CHOL, cholesterol; ALT, alanine aminotransferase; AST, aspartate aminotransferase; BUN, urea nitrogen; CRE, creatinine; PCC, prothrombin complex concentrate; CABG, coronary artery bypass grafting.

### 3.2. Relationship between PCC and venous thrombosis

As shown in Table 2, In the analysis without adjustment for confounders (model 1), perioperative PCC infusion significantly increased the risk of postoperative venous thrombosis (OR: 3.10, 95% CI: 1.26–7.59, *P *= 0.0135). In the model analysis adjusted for sex, age, and hospitalization days (model 2), perioperative PCC infusion was no longer significantly associated with the risk of postoperative venous thrombosis (OR: 1.76, 95% CI: 0.56–7.59, *P *= 0.3317). In the fully adjusted model (model 3), there was a marginally significant association between perioperative infusion of PCC and the risk of postoperative venous thrombosis (OR: 0.03, 95% CI: 0.00–1.23, *P *=* *0.0637).

**Table 2:** Association of PCC and usage of PCC with the risk of venous thrombosis.

	Model 1	Model 2	Model 3
	OR (95% CI, *P*)	OR (95% CI, *P*)	OR (95% CI, *P*)
PCC			
No	Reference	Reference	Reference
Yes	3.10 (1.26, 7.59) 0.0135	1.76 (0.56, 5.52) 0.3317	0.03 (0.00, 1.23) 0.0637
Usage of PCC			
No use	Reference	Reference	Reference
≥200–≤400 IU	2.43 (0.94, 6.30) 0.0673	1.67 (0.50, 5.58) 0.4038	0.02 (0.00, 11.54) 0.2231
>400–≤1,000 IU	4.33 (1.28, 14.70) 0.0186	1.35 (0.25, 7.26) 0.7232	0.00 (0.00, 1,901.40) 0.1966
>1,000–≤1,600 IU	7.43 (1.71, 32.29) 0.0075	3.97 (0.57, 27.82) 0.1646	0.00 (0.00, 1,997.47) 0.2700
*P* for trend	0.002	0.243	0.043

Model 1: no covariates were adjusted.

Model 2: Sex; Age; Hospitalization days.

Model 3: Sex; Age; Hospitalization days; Renal insufficiency; liver insufficiency; Central venous line placement; Hemoglobin; Red blood cell count; Platelet count; Activated partial thromboplastin time; D-Dimer; Total bilirubin; Indirect bilirubin; Albumin; Creatinine; Total duration of surgery; Surgery type; Total extracorporeal circulation admission; Red blood cells; Blood loss; Warfarin; Warfarin combined with heparin drugs; Warfarin combined with antiplatelet drugs; Postoperative renal insufficiency; Postoperative liver insufficiency; Postoperative ICU days.

OR, odds ratio; CI, confidence interval; PCC, Prothrombin complex concentrate.

Based on the recommended dosage in the indications for PCC, the weight of the Asian population, and the European consensus statement on the use of four-factor prothrombin complex concentrates in patients undergoing cardiac and non-cardiac surgery (23), the magnitude of PCC use was divided into four groups: no use, low dose, medium dose, and high dose, and trend analysis was conducted. In the unadjusted analysis (model 1), the risk of postoperative venous thrombosis increased significantly with increasing perioperative PCC usage (*p* for trend = 0.002). However, in model 2, this trend was not observed. In the fully adjusted model (model 3), this trend still exists (*p* for trend = 0.043).

### 3.3. Subgroup analysis

**Figure 1:**
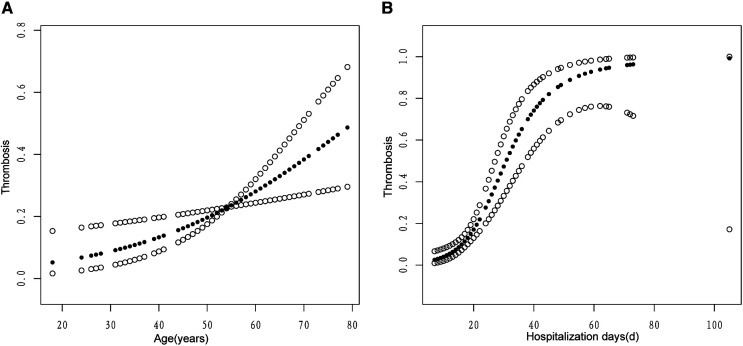
The associations of age (**A**) and hospitalization days (**B**) with venous thrombosis. A linear relationship was observed between age and hospitalization days with venous thrombosis. A solid circle line represents the smooth curve fit between variables. Hollow circle bands represent 95% of the confidence interval.

We determined the positive association between age and hospitalization days with venous thrombosis using a fitted smoothing curve (Figure 1). We used threshold effect analysis to determine the thresholds for age and hospitalization days. When the patient aged ≥66 years, the risk of venous thrombosis increases with age (OR: 1.16, 95% CI: 1.00–1.35, *P* = 0.0482), whereas the above was not statistically significant when the patient aged <66 years (OR: 1.03, 95% CI: 0.99–1.07, *P* = 0.1547). When the patient's hospitalization days ≤36 days, the risk of venous thrombosis increases with hospitalization days (OR: 1.20, 95% CI: 1.11–1.29, *P* < 0.0001), but when the patient's hospitalization days >36 days, the above is not statistically significant (OR: 1.04, 95% CI: 0.98–1.11, *P* = 0.2032).

The results of the stratified analysis are shown in (Table 3), in the analysis without adjustment for confounding variables (model 1), perioperative PCC infusion was associated with an increased risk of postoperative venous thrombosis in patients aged <66 years (OR: 3.52, 95% CI: 1.12–11.10, *P *= 0.0316); Patients not on warfarin, warfarin combined with heparin, warfarin combined with antiplatelet agents postoperatively, Perioperative PCC infusion is associated with a significantly increased risk of postoperative venous thrombosis (OR: 5.21, 95% CI: 1.35–20.14, *P *= 0.0167) (OR: 3.68, 95% CI: 1.14–11.86, *P *= 0.0289) (OR: 3.06, 95% CI: 1.17–8.00, *P *= 0.0227). However, in the analysis of the model adjusted for sex, age, and hospitalization days (model 2), this association above was not statistically different.

**Table 3:** Effect size of PCC on venous thrombosis in each subgroup.

	Cases	Controls	Model 1	Model 2
			OR (95% CI, *P*)	OR (95% CI, *P*)
Age (years)				
<66	77	47	3.52 (1.12, 11.10) 0.0316	1.80 (0.41, 7.82) 0.4338
≥66	25	12	2.36 (0.51, 10.85) 0.2710	1.47 (0.14, 15.25) 0.7465
Hospitalization days(d)				
≤36	81	53	3.48 (0.95, 12.77) 0.0599	1.58 (0.40, 6.29) 0.5180
>36	21	6	1.60 (0.22, 11.50) 0.6404	1.92 (0.24, 15.47) 0.5389
Warfarin				
No	42	26	5.21 (1.35, 20.14) 0.0167	3.40 (0.63, 18.30) 0.1541
Yes	60	33	2.01 (0.60, 6.74) 0.2607	0.57 (0.09, 3.60) 0.5489
Warfarin combined with heparin drugs				
No	62	40	3.68 (1.14, 11.86) 0.0289	2.20 (0.49, 9.90) 0.3041
Yes	40	19	2.29 (0.56, 9.33) 0.2493	0.91 (0.13, 6.30) 0.9238
Warfarin combined with antiplatelet drugs				
No	94	54	3.06 (1.17, 8.00) 0.0227	1.66 (0.48, 5.77) 0.4218
Yes	8	5	4.00 (0.30, 53.47) 0.2947	0.01 (0.00, 107.44) 0.3277

Model 1: no covariates were adjusted.

Model 2: Sex; Age; Hospitalization days.

## 4. Discussion

Cardiac surgery involves vital organs, long and traumatic operation time, inflammatory response, and the effects of extracorporeal circulation, which lead to patients' vulnerability to coagulation disorders. Venous thrombosis is a serious complication after cardiac surgery, but fewer studies are exploring the occurrence of postoperative venous thrombosis and the factors influencing it. A prospective study showed asymptomatic venous thromboembolism in 20%–41% of patients after cardiovascular surgery (24). Overall, the reported incidence of VTE post-CABG surgery varies widely, with estimates ranging from 0.4% to 41%, in part due to symptom driven vs. routine VTE screening methods, diagnostic modalities, and thromboprophylaxis measures (1, 25, 26). In China, the standardized prevention rate of venous thrombosis is included in the improvement goal of national medical quality and safety. A study in China (27) found that from 2007 to 2017, the incidence of venous thrombosis in surgical patients in China has been increasing year by year (1.4%–7.4%), but the mortality rate has shown a decreasing trend (4.8%–2.1%). In addition, through the evaluation of the risk of VTE in hospitalized patients and the implementation of preventive measures, it was found that there is a lack of risk management for venous thromboembolism, and it is necessary to continuously improve the awareness of doctors and the practice of VTE prevention and treatment measures (28). However, there are few studies on venous thrombosis after cardiac surgery in China. This is a retrospective case-control study, we enrolled 161 patients from more than 1,000 inpatients (including valve surgery, CABG, etc.) who received cardiac surgery in our cardiovascular surgery in 2021, of whom 23% had symptomatic or Asymptomatic venous thrombosis (all peripheral venous thrombosis, no pulmonary embolism), to explore the relationship between perioperative use of PCC and postoperative venous thrombosis in patients undergoing cardiac surgery. Our findings showed that perioperative PCC infusion significantly increased the risk of postoperative venous thrombosis in an analysis unadjusted for confounders. In model analyses adjusted for sex, age, and hospitalization days, perioperative PCC infusion was no longer significantly associated with the risk of postoperative venous thrombosis. In the fully adjusted model, there was a marginally significant association between perioperative infusion of PCC and the risk of postoperative venous thrombosis. In the unadjusted analysis (model 1), the risk of postoperative venous thrombosis increased significantly with increasing perioperative PCC usage (*p* for trend = 0.002) in four groups: no use, low dose, medium dose, and high dose, according to the amount of PCC used. However, in model 2, this trend was not observed. In the fully adjusted model (model 3), this trend still exists.

Patients may experience significant blood loss during cardiac surgery. Approximately 10% of all patients undergoing cardiac surgery suffer from severe or massive blood loss, and up to 5% require urgent re-exploration to correct ongoing bleeding and establish adequate hemostasis (29, 30). We included 3.1% of patients in the study who underwent re-exploration, 5.4% in the case group, and 2.4% in the control group, respectively. The rate of re-exploration for bleeding was slightly lower than reported in the literature. PCC can supplement coagulation factor deficiency in surgical patients and can initiate exogenous coagulation and initiate clotting via the bypass activation pathway. PCC offers several advantages in the treatment of cardiac surgery-related diseases, such as faster transfusion, lower volume overload, shorter preparation time, no need for blood type matching, and lower transfusion response rate (31). Several retrospective studies have shown less perioperative bleeding and transfusion in patients with PCC compared to fresh frozen plasma (FFP). In an observational study, fewer red blood cells were transfused compared to FFP when PCC was given as the first-line treatment for bleeding after CPB (10). Studies have analyzed perioperative bleeding and transfusion outcomes in patients undergoing cardiac surgery treated with PCC or plasma (32). The results of this study suggest that patients receiving PCC are more likely to avoid allogeneic transfusions.

Venous thrombosis after cardiac surgery can lead to serious complications, including pulmonary embolism, stroke, bleeding, heart failure, and even death. A study found that adult cardiac surgery patients with thrombosis had a 68%–126% increase in hospital length of stay (33). Our study also showed that the length of stay of patients in the case group was longer than that of the control group (42.7 ± 18.3 vs. 18.8 ± 10.5, *P* < 0.001), and 16.2% of patients in the case group had other thrombotic events, such as left atrial thrombus or left ventricular thrombus. The major safety concerns with PCC are related to thrombotic events such as stroke, myocardial infarction, pulmonary embolism, diffuse intravascular coagulation, and deep vein thrombosis. Our study showed that in the control group, 2 patients underwent cardiac surgery due to valve thrombosis. The use of PCC after cardiac surgery did not increase the formation of graft or valve thrombosis in patients, but venous thrombosis led to longer hospital stays and increased medical costs. There is little evidence regarding the association of PCC use in cardiac surgery with thrombotic events. A case of a patient who developed massive intracardiac and pulmonary artery thrombosis after a complex direct cardiac surgery with the infusion of moderate doses of PCC (19). Although thrombotic events are associated with PCC administration, in most cases they can be attributed to the patient's underlying thrombotic risk factors. Furthermore, the low incidence of such adverse events has been further reduced over the past few years due to recent improvements in the composition of PCC (i.e., the addition of coagulation inhibitors, reduced use of active clotting factors, and improved balance of clotting factors).

Our study also showed that perioperative PCC infusion significantly increased the risk of postoperative venous thrombosis when no confounding factors were taken into account. Because of the many factors that influence venous thrombosis, perioperative PCC infusion was not significantly associated with postoperative venous thrombosis in a fully adjusted model when possible confounding factors were considered. Much of the current evidence also suggests that perioperative use of PCC is safe and effective and does not increase thromboembolic events or mortality. A recently published literature mentioned “In the massively bleeding patient with coagulopathy, our group recommends the administration of an initial bolus of 25 IU.kg^−1^. In patients with a high risk for thromboembolic complications, e.g., cardiac surgery, the administration of an initial half-dose bolus (12.5 IU.kg^−1^) should be considered” (23). Our data also show that perioperative PCC infusion is not significantly associated with the occurrence of postoperative venous thrombosis in all subgroups analyzed, after careful model adjustment. These inconsistent results may be due to differences between studies, including study populations, study design, and controlling for confounding factors.

Many factors influence venous thrombosis, A study discussed the risk factors for venous thrombosis from an epidemiological point of view and summarized the risk factors affecting the first occurrence of venous thrombosis including antithrombin deficiency, increasing age, malignancy, chronic kidney disease, oral contraceptives, surgery, etc (34). We determined a positive association between age and hospitalization days with the occurrence of venous thrombosis using a fitted smoothing curve. A study statistically analyzed the recurrence rate of VTE in different age groups, where 10.5% of the patients were older than 70 years and 61.8% were between 40 and 70 years, showing no statistically significant difference in the recurrence rate in different age groups (35). The investigation found that age was associated with the risk of recurrence of venous thromboembolism (36). This is consistent with our findings, this may be due to decreased vascular elasticity, slow blood flow, increased number of multiple coagulation factors, and decreased function of the fibrinolytic system with age in the elderly (37–39). Perioperative use of PCC, combined with reduced activity after cardiac surgery, in turn, predisposes elderly patients to thrombosis. At the same time, many elderly people have a combination of hypertension, diabetes mellitus, and hyperlipidemia, which damage the vascular endothelium and contribute to intravascular thrombosis.

For valve surgery, to prevent the formation of valve thrombosis, anticoagulant drugs should be used 24–48 h after surgery, taking into account the patient's drainage flow rate, drainage fluid color, coagulation parameters, etc. Patients with CABG need to be given antiplatelet therapy because of Atherosclerosis blocking blood vessels. In this study, patients with valve surgery and CABG were given anticoagulants and antiplatelet drugs according to the coagulation condition, such as unfractionated heparin, low-molecular-weight heparin, warfarin, aspirin, clopidogrel, and Warfarin was the most commonly used oral anticoagulant (40, 41). Warfarin has a long half-life, slow onset of action, inhibits II, VII, IX, and X along with protein C and S synthesis, has a transient procoagulant effect at the beginning of drug administration, and increases the number of factors II, VII, IX, and X in the blood with perioperative use of PCC, which may increase the risk of thrombosis if anticoagulation therapy is not overlapped with parenteral anticoagulants at the beginning. In addition, studies have shown a high incidence of major bleeding and clinically relevant non-major bleeding events in elderly patients treated with anticoagulation for venous thromboembolism, and these bleeding events create a significant burden of disease (42). Our study also showed an increase in other bleeding events in the case group compared to the control group (8.1% vs. 1.6%). In addition, the hospitalization expenses of the case group were significantly higher than those of the control group ($75,135.9 ± 39,048.5 vs. $25,466.4 ± 13,163.7). High-dose heparin, protamine antagonism, inflammatory reaction, and activation of the fibrinolytic system may aggravate coagulation disorder in patients undergoing cardiac surgery. The use of heparin and protamine in the case group and the control group were (201.4 ± 96.9 mg vs. 189.3 ± 56.1 mg, 294.9 ± 139.3 mg vs. 271.6 ± 79.9 mg), respectively, and there was no significant difference between the two groups. Therefore, the timing of anticoagulation therapy given by physicians is more prudent. Long-term hospitalization also carries a higher risk of venous thromboembolism (43). This is consistent with our fitted curve results, patients with long hospital stays tend to have longer surgical times, more severe disease, more comorbidities, poorer prognosis, and a higher risk of venous thrombosis. In addition, the use of PCC in the perioperative period of liver transplantation is independently associated with the primary composite endpoints of hepatic artery thrombosis, portal vein thrombosis, and inferior vena cava thrombosis (44). However, intraoperative use of PCC in cardiac surgery resulting in valve or vascular graft thrombosis has not been reported. Leading to acute kidney injury or venous thrombosis has been reported (10). The patients included in our study did not develop graft thrombosis, on the one hand, due to the one-time intraoperative use of PCC at a dose between 200 and 1,600 IU, which is not a high-dose repeated use (23). The half-lives of factors II, VII, IX, and X contained in PCC are 42–72 h, 4–6 h, 21–30 h, and 27–48 h, respectively, which can make up for the consumption of coagulation factors by the patients intraoperatively, and reduce the risk of postoperative bleeding, but large amounts can lead to thrombotic events. Therefore, studies with larger sample sizes are needed to determine the appropriate dose of PCC to use.

The coagulation status of patients during cardiac surgery is altered (45), in which coagulation factors are lacking, such as direct destruction of coagulation factors by contact with non-biological materials, a relative reduction of coagulation factor concentrations by hemodilution, and inhibition of platelet and various coagulation factor activities due to hypothermia. This is characterized by a reduced ability to synthesize coagulation and anticoagulation factors as well as fibrinolytic and antifibrinolytic factors, and an imbalance of these factors can lead to thrombosis and bleeding (46). The attending physician provides PCC empirically based on the patient's intraoperative bleeding volume, extracorporeal circulation time, and TEG. Larger sample sizes need to be validated for the specific dose and timing of PCC use. For perioperative bleeding, clinicians managing patients should be aware of the importance of the mechanistic cascade and multimodal approach of PCC to affect coagulation, as well as the management of diffuse bleeding associated with surgery and the use of PCC in moderation.

The limitations of this study are as follows: (1) Our study used a case-control design rather than a cohort design, which would have been a more robust approach. Because this is a single-center study, the generalizability and external validity of the results may be limited. Meanwhile, there was a significant difference in coagulopathy between the two groups after cardiac surgery, with the case group and the control group (10.8% vs. 1.6%, *p *= 0.01), which could be another major confounder. Continued case collection and expansion of the sample size will be needed in future studies. Therefore, future large-scale, multicenter, prospective studies are needed to accurately identify and improve factors for adverse outcomes and further studies to determine whether the infusion of PCC provides benefit to patients. (2) The criteria for the surgeon to decide whether to use PCC include hemoglobin levels, coagulation indicators, and bleeding after protamine antagonism. Although thromboelastography or ROTEM can provide a deeper understanding of coagulation factor deficiency and guide the use of exogenous factor substitution, these tools were not used by everyone in the study patient population during the study period, and due to limitations in electronic medical record informatization, intraoperative related indicators were not fully digitized, and a small amount of data was not obtained. Cardiac surgery employing CPB and deep hypothermic circulatory arrest can induce coagulation disturbances and bleeding complications (47). However, studies have shown that hypothermic circulatory arrest does not induce coagulopathy *in vitro*. In the mock circulation loop (MCL) study, MCL decreases coagulation due to the deterioration of platelets (48). Again, due to the lack of hypothermic circulatory arrest data, this factor has not been further explored (3). The population included in this study was a single use of PCC, with doses ranging from 200 to 1,600 IU. The dosage of PCC was not standardized, and some patients received doses lower than the standard dosage strategy (23), as well as a reversal dose of 25–50 IU.kg^−1^ of warfarin based on INR. As of discharge, no graft thrombosis was found among the included patients in this article. Research has reported that the use of PCC increases graft thrombosis, which may be related to high doses and multiple uses. Although postoperative VTE formation after cardiac surgery may lead to increased patient healthcare costs and length of hospital stay due to fatal pulmonary embolism or serious sequelae, valve thrombosis and graft thrombosis due to PCC can lead to more serious medical accidents, and future studies on the safety of PCC need to include this as a study endpoint.

In conclusion, our study found that a single intraoperative use of a certain dose range of PCC (200–1,600 IU) during cardiac surgery was not significantly associated with the development of venous thrombosis after cardiac surgery. With the current data, further rigorous randomized controlled trials of PCC on venous thrombosis after cardiac surgery are needed.

## Data Availability

The raw data supporting the conclusions of this article will be made available by the authors, without undue reservation.
